# The prognostic significance of Cdc6 and Cdt1 in breast cancer

**DOI:** 10.1038/s41598-017-00998-9

**Published:** 2017-04-20

**Authors:** Ravikiran Mahadevappa, Henrique Neves, Shun Ming Yuen, Yuchen Bai, Cian M. McCrudden, Hiu Fung Yuen, Qing Wen, Shu Dong Zhang, Hang Fai Kwok

**Affiliations:** 1 Faculty of Health Sciences, University of Macau, Avenida de Universidade, Taipa, Macau SAR China; 2 School of Pharmacy, Queen’s University Belfast, Belfast, UK; 3 Institute of Molecular and Cell Biology, A*STAR, Singapore, Singapore; 4 Centre for Cancer Research & Cell Biology and Centre for Public Health, School of Medicine, Dentistry and Biomedical Sciences, Queen’s University Belfast, Belfast, UK; 5 Northern Ireland Centre for Stratified Medicine, Biomedical Sciences Research Institute, Ulster University, Londonderry, UK

## Abstract

DNA replication is a critical step in cell proliferation. Overexpression of MCM2-7 genes correlated with poor prognosis in breast cancer patients. However, the roles of Cdc6 and Cdt1, which work with MCMs to regulate DNA replication, in breast cancers are largely unknown. In the present study, we have shown that the expression levels of Cdc6 and Cdt1 were both significantly correlated with an increasing number of MCM2-7 genes overexpression. Both Cdc6 and Cdt1, when expressed in a high level, alone or in combination, were significantly associated with poorer survival in the breast cancer patient cohort (n = 1441). In line with this finding, the expression of Cdc6 and Cdt1 was upregulated in breast cancer cells compared to normal breast epithelial cells. Expression of Cdc6 and Cdt1 was significantly higher in ER negative breast cancer, and was suppressed when ER signalling was inhibited either by tamoxifen *in vitro* or letrozole in human subjects. Importantly, breast cancer patients who responded to letrozole expressed significantly lower Cdc6 than those patients who did not respond. Our results suggest that Cdc6 is a potential prognostic marker and therapeutic target in breast cancer patients.

## Introduction

DNA replication is an all-or-nothing process; once DNA replication begins, it proceeds to completion and a DNA segment is never replicated twice in one cell cycle^1^. The precision of timely initiation of DNA replication is very important for preventing abnormal inheritance of genomic pool to daughter cells. To avoid undesirable consequences such as under or over DNA replication, the entire process is tightly regulated by a multi-subunit initiator protein complex known as “Pre-replication complex (pre-RC)” or “Licensing complex”. This complex consists of origin-recognition complex (ORC; consists of Orc1 to 6), protein Cell division cycle – 6 (Cdc6), protein Chromatin Licensing and DNA Replication Factor 1 (Cdt1) and Minichromosome maintenance proteins (MCMs). Establishment of the regulatory process requires a stepwise assembly of ORC, CDC6, CDT1 and MCMs in the replication origin^1^.

During late M phase and early G1 phase of the cell cycle, ORC binds to the DNA replication origin which acts as a platform for recruiting Cdc6 and Cdt1^2^, which then recruits MCMs onto the origin for initiation of DNA replication. When the MCMs move on the chromatin as elongation proceeds, the origin is then converted to an unlicensed state by the binding of Geminin to Cdt1 to prevent DNA re-replication^1, 3^. Other than Geminin, cyclin-dependent kinases (CDKs) also play an important role in regulating the initiation of DNA replication. CDK activity increases from the onset of S-phase to M-phase leading to phosphorylation of licensing factors to prevent re-licensing^1, 3^. After DNA duplication and chromosome segregation have been completed, CDKs are then inactivated and Geminin is degraded to prepare for a new round of DNA replication^1^. Genetic alterations leading to deletion or overexpression of these proteins have severe consequences on genomic stability and cell proliferation. Deletion of either Cdc6 or Cdt1 prevents normal association of MCMs with chromatin during G1 phase thereby stalling cell cycle progression^4^. In-contrast, over-expression of Cdt1 is observed to over-ride cell control checkpoints initiating DNA re-replication through activation of ATM/ATR checkpoint pathways^5^.

Overexpression of Cdc6 and Cdt1 has been shown to contribute to oncogenesis^6, 7^ and their upregulations are linked to cancer progression in various types of cancer^8–13^. Cdc6 has been shown to be regulated by estrogen^14^, and is downregulated in methionine-mediated inhibition of cell proliferation^15^. However, little is known regarding the prognostic significance of Cdc6 and Cdt1 in breast cancer.

We have previously shown that MCMs play an important role in breast cancer progression and that the over-expression of multiple MCMs is significantly correlated with poor prognosis in breast cancer patients^16^. These results, together with others indicate that MCMs contribute to the development and progression of breast cancer^17–20^, support the hypothesis that increased expression of genes correlated with DNA replication licensing may be a prognostic marker and a therapeutic target for breast cancer^21^. Since Cdc6, Cdt1 and Orc1 work cooperatively with MCM2-7 to initiate DNA replication, in our current study we have investigated whether there are associations between these three genes and clinicopathological parameters or expression of MCMs in breast cancer.

## Results

### The association between expressions of MCM2-7, Cdc6, Cdt1 and Orc1 in breast cancer specimens

**Figure 1:**
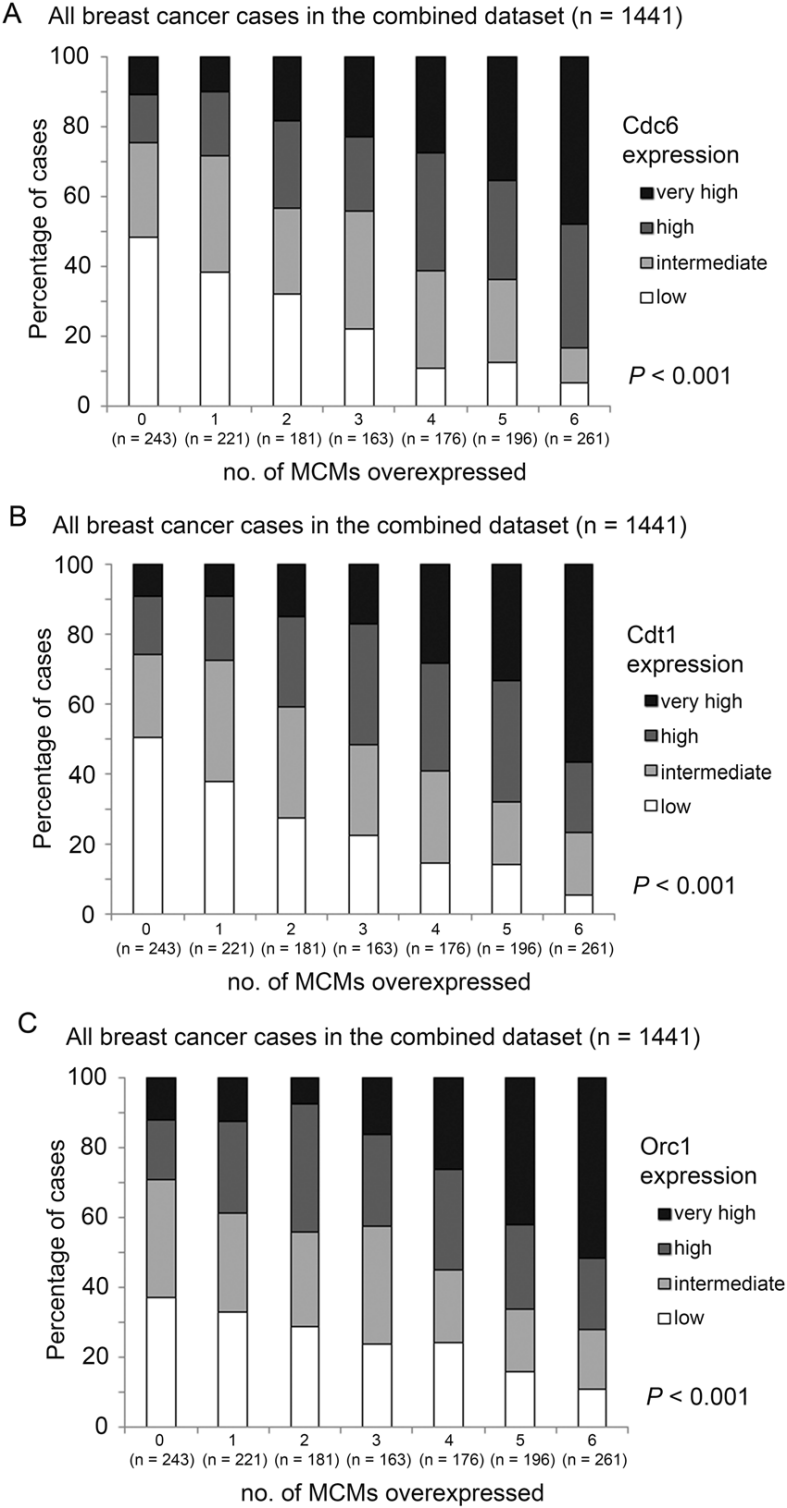
The expression levels of Cdc6, Cdt1 and Orc1 in breast cancer patients expressing various levels of MCM genes. Histograms showing percentage of specimens that expressed different levels of (**A**) Cdc6, (**B**) Cdt1 and (**C**) Orc1 in six groups of specimens stratified by the number of MCM2-7 genes they overexpress.

Previously, we have shown that overexpression of increasing numbers of MCM2-7 genes is associated with poor prognosis in breast cancer patients. Since Cdc6, Cdt1 and Orc1 work cooperatively with MCM2-7 to initiate DNA replication, we have investigated whether there are associations between the numbers of overexpressed MCM2-7 genes and expression of these three genes in breast cancer specimens. There was a statistically significant positive correlation between number of MCM2-7 genes expressed at a high level and Cdc6 expression level (Spearman’s rank test, r = 0.435, p < 0.001; Fig. 1A). A similar pattern of correlation was observed when Cdt1 expression level was stratified with the number of MCM2-7 genes expressed at high level (Spearman’s rank test, r = 0.448, p < 0.001; Fig. 1B). There was also a significant positive correlation between Orc1 expression and number of MCM2-7 genes expressed at a high level in breast cancer specimens (spearman’s rank test, r = 0.349, p < 0.001; Fig. 1C).

**Figure 2:**
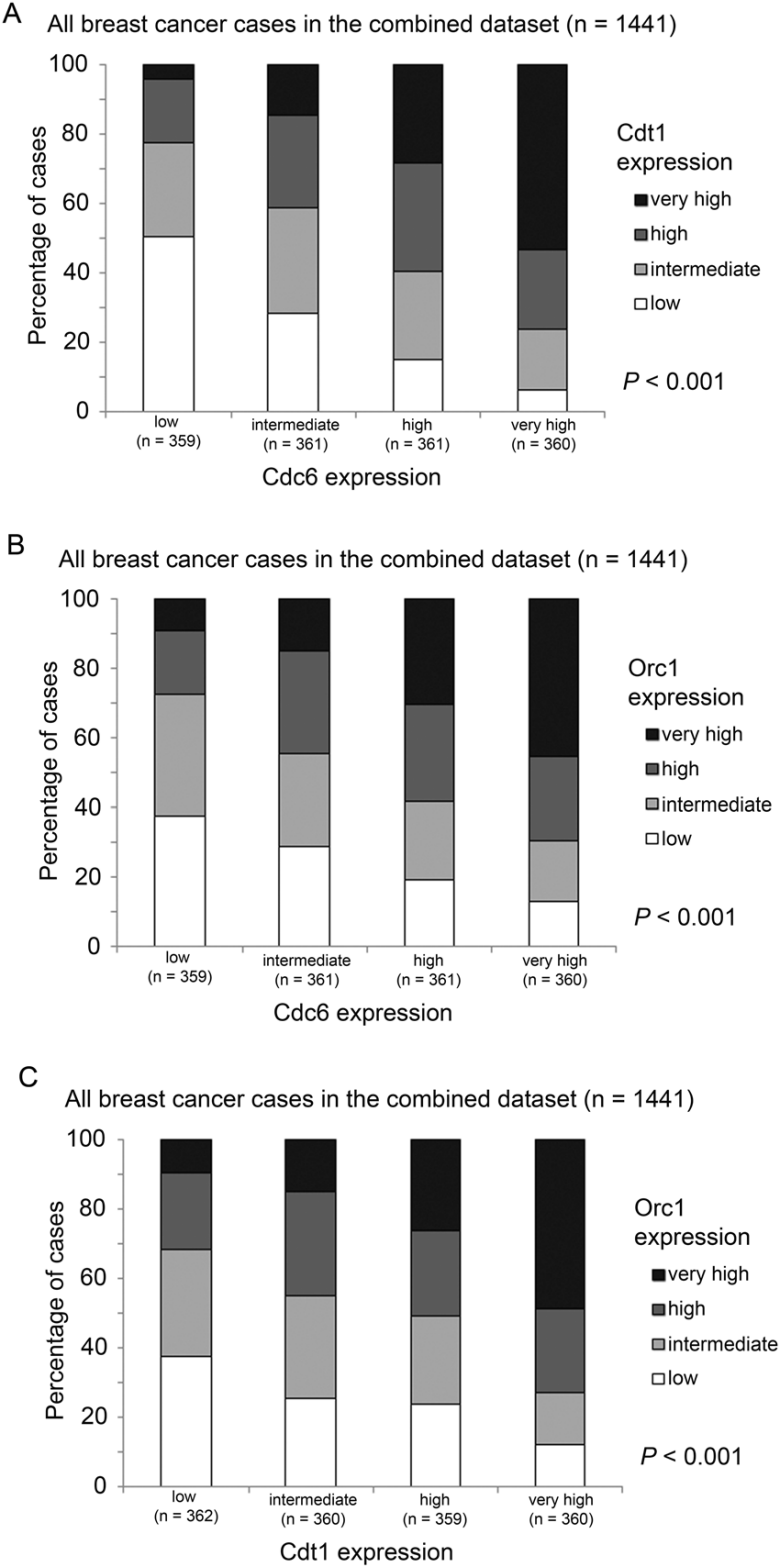
The associations between the expression levels of Cdc6, Cdt1 and Orc1 in breast cancer specimens. Histograms showing percentage of specimens that expressed different levels of (**A**) Cdc6 vs. Cdt1, (**B**) Cdc6 vs. Orc1 and (**C**) Cdt1 and Orc1, in breast cancer specimens.

Indeed, we also found a significantly positive correlation among Cdc6, Cdt1 and Orc1 expression levels. Expression of Cdc6 was significantly positively correlated with Cdt1 expression (Spearman’s rank test, r = 0.485, p < 0.001; Fig. 2A) and Orc1 expression (Spearman’s rank test, r = 0.348, p < 0.001; Fig. 2B). Similarly, a significantly positive correlation was observed for the expression levels of Cdt1 and Orc1 (r = 0.338; p < 0.001; Fig. 2C).

### The association between the expression levels of Cdc6 and Cdt1 and breast cancer patient survival

**Figure 3:**
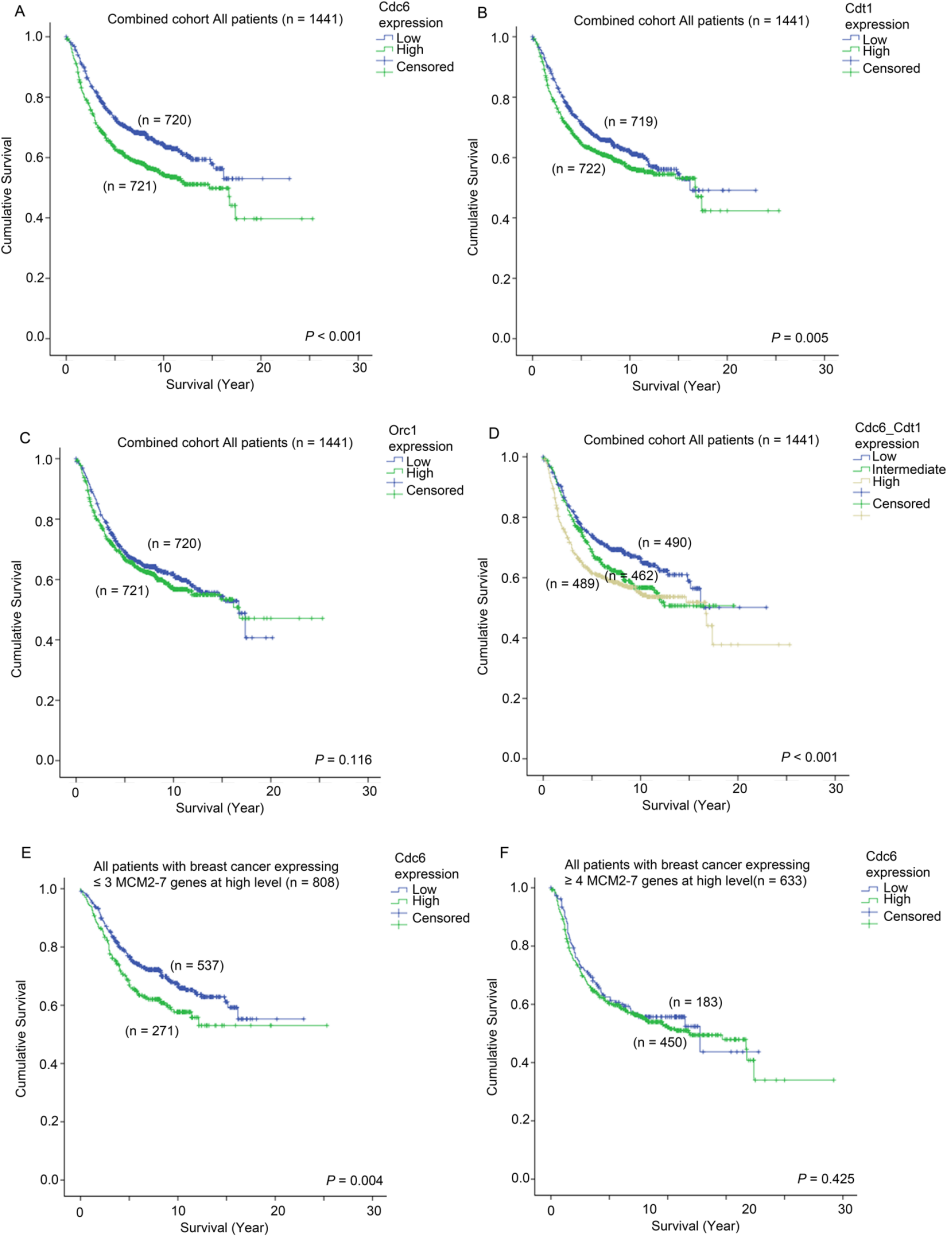
Survival analysis of patients who express different levels of Cdc6, Cdt1 and Orc1. Kaplan-Meier curves showing survival time of patients who express different levels of (**A**) Cdc6, (**B**), Cdt1 and (**C**) Orc1 in the combined breast cancer patient cohort. (**D**) Kaplan Meier curves showing survival time of patients who express different levels of Cdc6 and Cdt1 analyzed in combination in the whole breast cancer patient cohort. Kaplan Meier curves showing survival time of patients expressing different levels of Cdc6 in breast cancer cohort stratified based on the number of MCM genes they overexpress (E ≤ 3 MCMs overexpressed) and (F ≥ 4 MCMs overexpressed).

We have previously reported a correlation between increasing numbers of MCM genes being overexpressed and poor prognosis in breast cancer patients^16^. Here, we investigated whether Cdc6, Cdt1 or Orc1 also confer prognostic value to breast cancer patients. In the combined cohort as described previously^16^, patients whose breast cancer expressed a low level of Cdc6 had a significantly longer survival than those patients whose tumors expressed Cdc6 at a high level (Wilcoxon-Gehan test, p < 0.001; Univariate Cox regression, hazard ratio = 1.409, 95% confidence interval = 1.192–1.666, p < 0.001; Fig. 3A). Similarly, patients whose tumors expressed a low level of Cdt1 had a significantly longer survival time than those whose tumors expressed a high level of Cdt1 (Wilcoxon-Gehan test, p = 0.005; Univariate Cox regression, hazard ratio = 1.199, 95% confidence interval = 1.016–1.416, p = 0.032; Fig. 3B). The associations between expression level and survival was much obvious for CDC6, but less so for Cdt1. Conversely, Orc1 expression was not similarly predictive of survival in the combined cohort (Wilcoxon-Gehan test, p = 0.239; Univariate Cox regression, hazard ratio = 1.105, 95% confidence interval = 0.936–1.304, p = 0.241; Fig. 3C).

**Figure 4:**
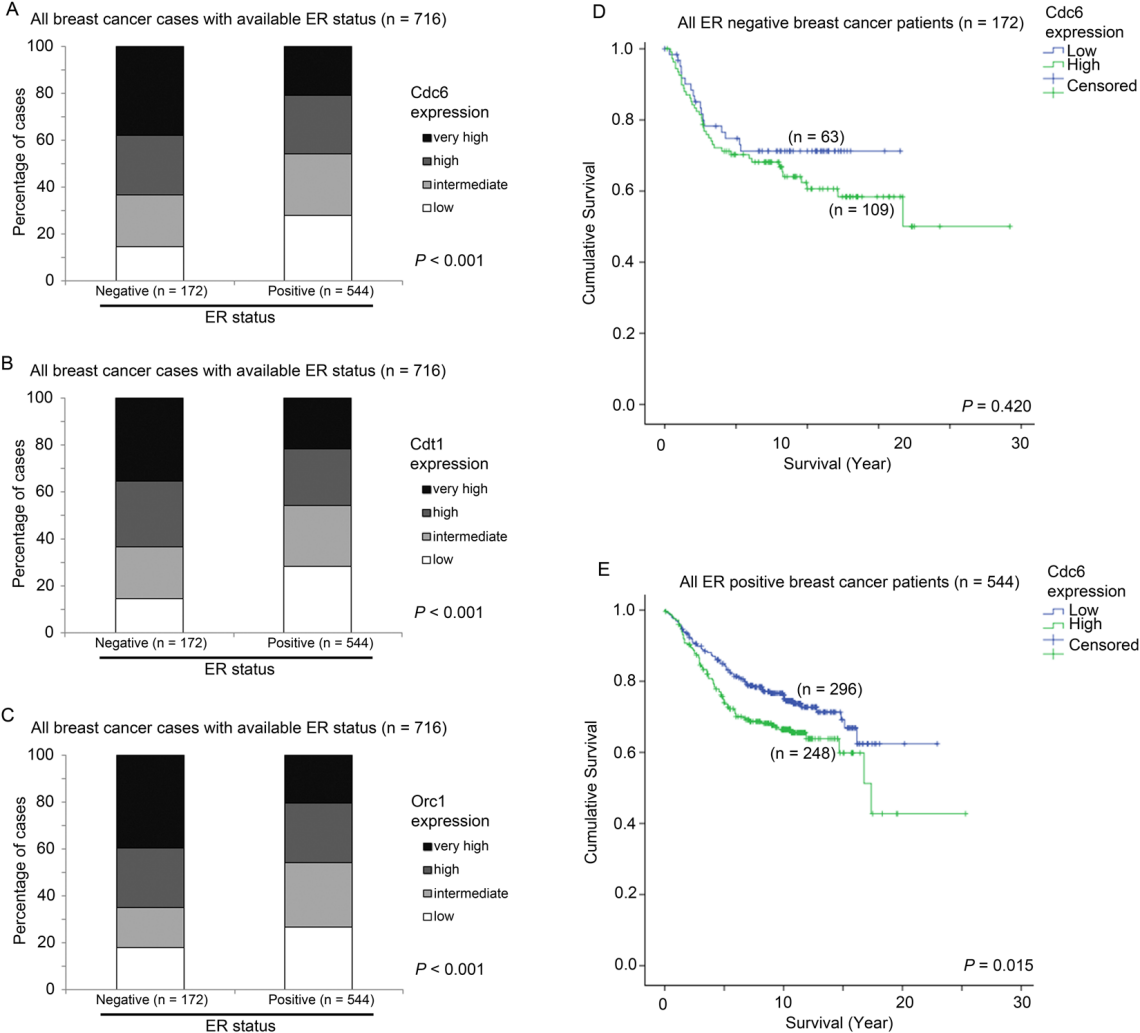
The association between expression of Cdc6, Cdt1 and Orc1 with different ER status and survival. Histograms showing the percentage of patients with various expression levels of (**A**) Cdc6, (**B**) Cdt1 and (**C**) Orc1 in patients stratified by ER status. Kaplan-Meier curves showing survival time of patients who express different levels of Cdc6 in patients with (**D**) ER-negative breast cancer and (**E**) ER-positive breast cancer.

Interestingly, when we considered Cdt1 and Cdc6 together, patients whose breast cancer expressed both Cdt1 and Cdc6 at a high level had a significantly shorter survival time compared to those patients whose breast cancer expressed only one of Cdc6 or Cdt1 at a high level (Wilcoxon-Gehan test, p = 0.01), or those patients whose breast cancer expressed both Cdc6 and Cdt1 at a low level (Wilcoxon-Gehan test, p < 0.001; Fig. 3D). Patients whose tumors expressed either Cdc6 or Cdt1 at a high level also had a shorter survival time compared to those whose cancer expressed both Cdc6 and Cdt1 at a low level (Wilcoxon-Gehan test, p = 0.052; Fig. 4D). Patients with either high tumoral Cdc6 or Cdt1 had a significantly higher risk of disease progression or death than those patients whose tumors expressed both genes at a low level (univariate Cox regression analysis; Hazard ratio = 1.259, 95% CI = 1.019–1.555, p = 0.033); this relationship was even more significant when both Cdc6 and Cdt1 were highly expressed (Hazard ratio = 1.481, 95% confidence interval = 1.206–1.818, p < 0.001).

More importantly, we also found that the prognostic significance of Cdc6 expression in the breast cancer patient cohort was independent of the prognostic significance of MCM2-7 genes. In a multivariate Cox-regression analysis using forward condition stepwise approach including MCM2-7 genes, Cdc6, Cdt1, Orc1 as variable factors, we found that both the number of MCM2-7 genes expressed at high level (Hazard ratio = 1.538, 95% confidence interval = 1.303–1.816, p < 0.001) and Cdc6 (Hazard ratio = 1.419, 95% confidence interval = 1.184–1.699, p = 0.025) were independent prognostic factors. We demonstrated in our previous report that patients whose tumors express more than three MCM2-7 genes at high level had a poorer prognosis compared to other patients^16^. Indeed, Cdc6 expression was significantly correlated with survival in patients whose cancer expressed three or fewer MCM2-7 genes at a high level (Wilcoxon-Gehan test, p = 0.004; Fig. 3E), but not in those patients whose cancer expressed four or more MCM2-7 genes at high level (p = 0.425; Fig. 3F). Further investigation is required to confirm the interaction between the expressions of CDC6 and MCMs in breast cancer.

**Figure 5:**
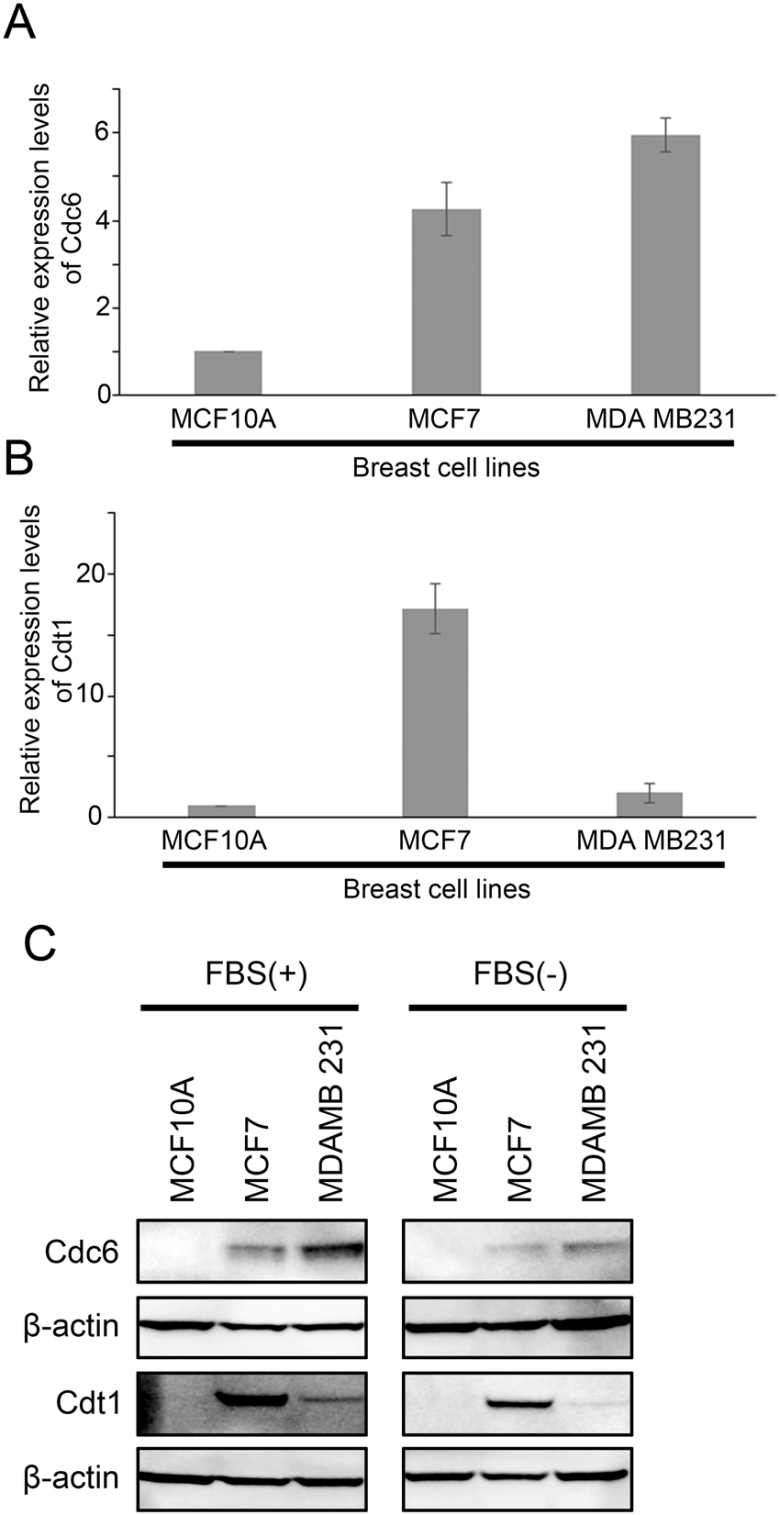
The expression of Cdc6 and Cdt1 in breast cell lines. Histograms showing (**A**) Cdc6 and (**B**) Cdt1 mRNA expression as measured by qPCR in normal breast epithelial cell line MCF10A, and two breast cancer cell lines, MCF7 and MDA MB231 in medium without Fetal Bovine Serum (FBS). (**C**) Western blot analysis of Cdc6, Cdt1 and β-actin in MCF10A, MCF7 and MDA MB231 cells in normal culture media (left panel) and in media without Fetal Bovine Serum (FBS; right panel).

We further tested the expression level of Cdc6 and Cdt1 in three breast cancer cell lines; three breast cell lines were chosen based on their aggressiveness and ER-positivity as well as their popularity in the literature. A normal breast epithelial cell line MCF10A and two malignant breast cancer cell lines, MCF7 and MDA MB231. We found that the mRNA expression levels of Cdc6 and Cdt1 were both higher in the two cancer cell lines compared to the normal breast epithelial cell line (Fig. 5A and B for Cdc6 and Cdt1, respectively). Similar results were obtained in their protein expression levels. As shown in Fig. 5C, expression levels of these two proteins were higher in the two cancer cell lines compared to the normal cells. Importantly, the expression of these two genes was correlated with proliferative signal. We found that in both MCF7 and MDA MB231 cells, withdrawal of FBS from the medium resulted in suppression of Cdc6 and Cdt1 expression (Fig. 5C).

### The association between expression levels of Cdc6, Cdt1 and Orc1 with ER status and ER signalling

Since Estrogen Receptor (ER) plays a significant role in breast cells proliferation, the associations between ER status and ER signalling and expression of Cdc6 and Cdt1 were further investigated. Of the 1441 breast cancer patients in the combined cohort, the ER status of 716 were known (172 cases – ER-negative, 544 cases – ER-positive). ER negative breast cancer specimens had a higher level expression of Cdc6 compared to those specimens with positive ER status (Chi Square test, p < 0.001; Fig. 4A). Similar results were observed for Cdt1 (Chi Square test, p < 0.001; Fig. 4B) and Orc1 (Chi Square test, p < 0.001; Fig. 4C).

Interestingly, the association between high Cdc6 expression and patient survival was only observed in ER-positive breast cancer patients (Wilcoxon-Gehan test, p = 0.420; Fig. 4D), but not in ER-negative breast cancer (Wilcoxon-Gehan test, p = 0.004; Fig. 4E).

### The association between expression level of Cdc6 and Cdt1, and the response to letrozole

We went on to investigate the impact of inhibited ER signalling on the expression of Cdc6 and Cdt1 in the ER-positive MCF7 breast cancer cells and human breast cancer specimens.

**Figure 6:**
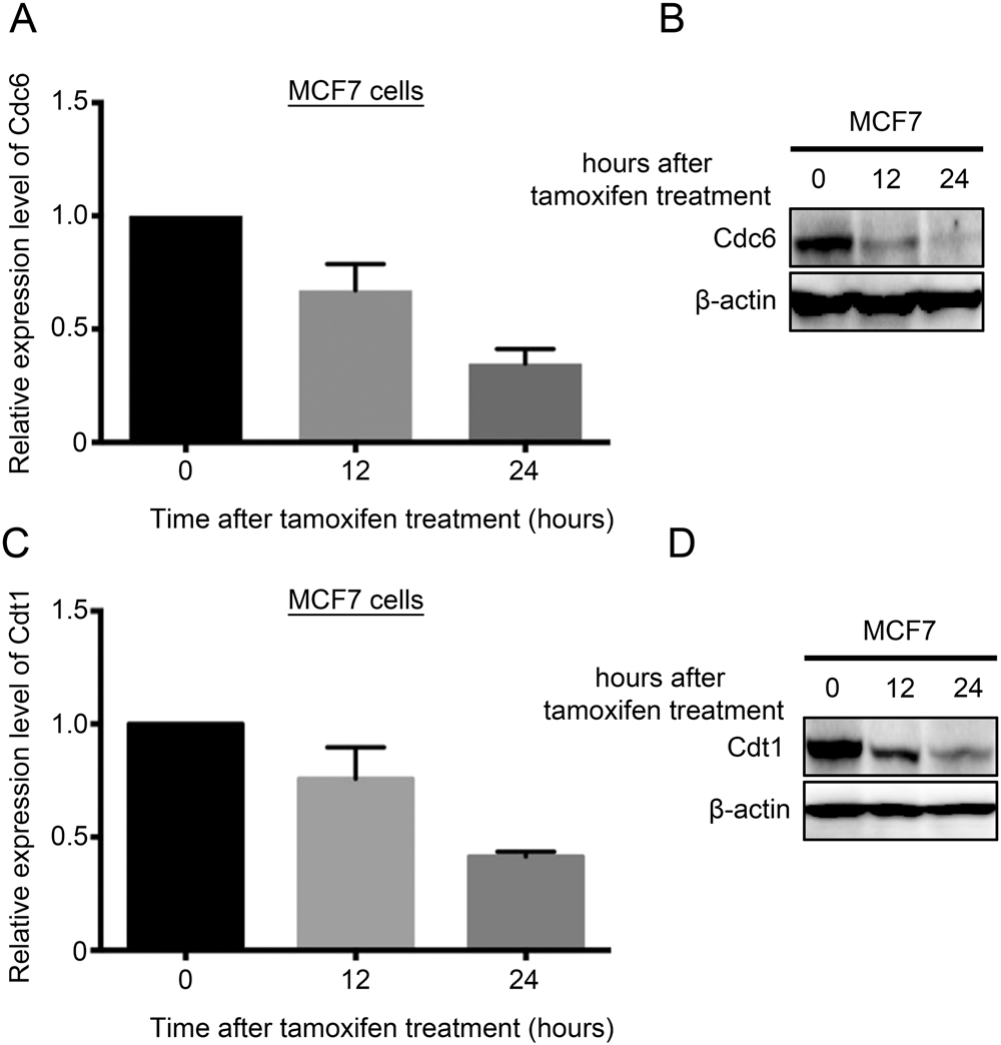
The expression of Cdc6 and Cdt1 upon inhibition of ER signalling. (**A**) Histogram and (**B**) Western blot showing the expression of Cdc6 in MCF7 cells treated with tamoxifen at 0, 12 and 24 hours post-treatment. (**C**) Histogram and (**D**) Western blot showing the expression of Cdt1 in MCF7 cells treated with tamoxifen at 0, 12 and 24 hours post-treatment.

In MCF7 cells, both mRNA and protein expressions of Cdc6 were significantly reduced by treatment with tamoxifen, an ER inhibitor, for 12 and 24 hours (Fig. 6A and B). Similar results were observed for Cdt1 (Fig. 6C and D). These results suggest that inhibition of ER signalling resulted in suppression of Cdc6 and Cdt1 expression in breast cancer cells.

**Figure 7:**
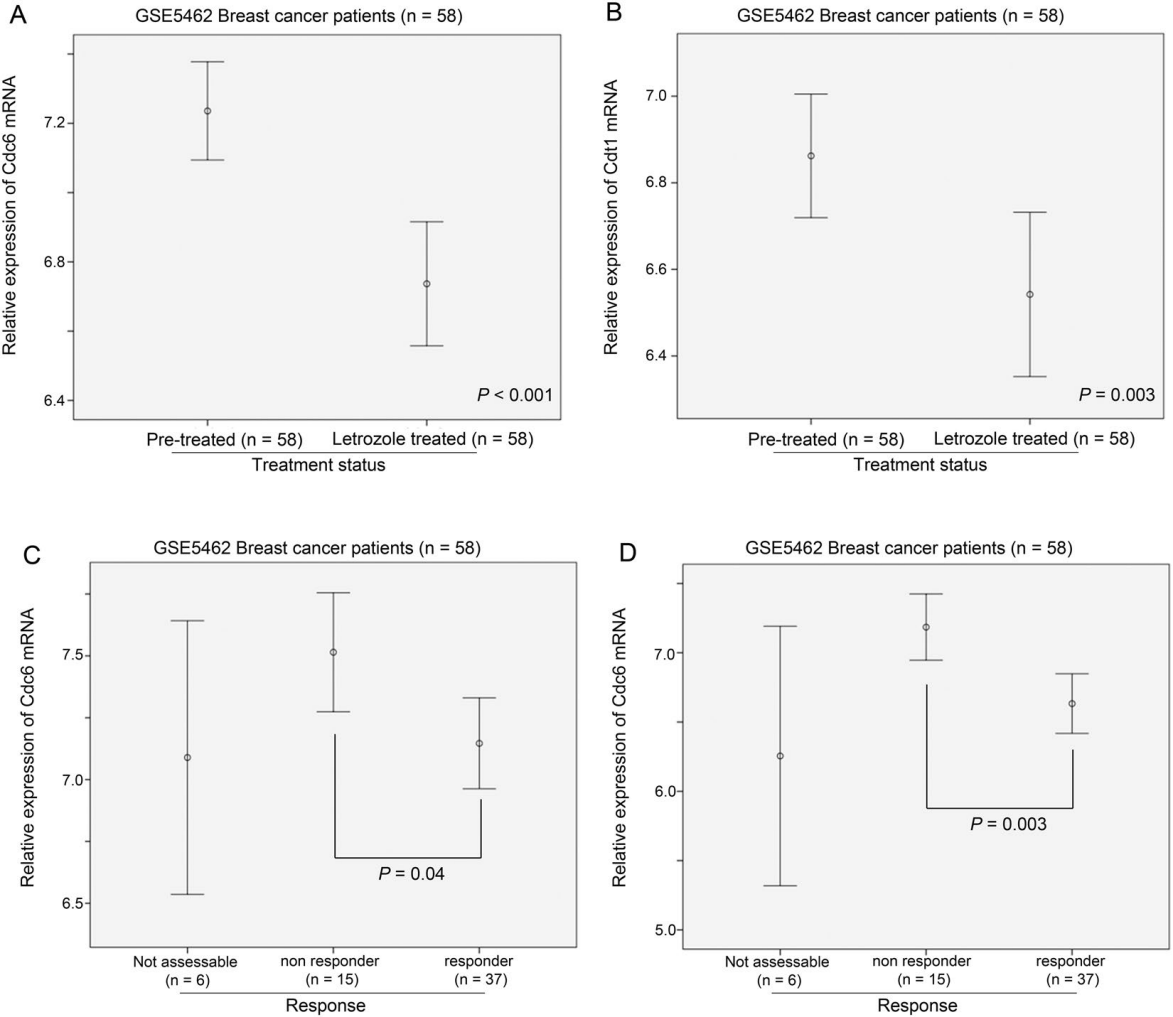
The expression of Cdc6 and Cdt1 in breast cancer specimens treated with letrozole. Error plots showing the expression levels of (**A**) Cdc6 and (**B**) Cdt1 in breast cancer specimens pre- and post-treatment with letrozole. Error plots showing the impact of ER signalling inhibition on the expression of Cdc6 in breast cancer specimens (**C**) pre-treatment and (**D**) post-treatment with letrozole.

To confirm these *in vitro* findings, we investigated the impact of letrozole treatment on the expression levels of Cdc6 and Cdt1 in breast cancer. From a dataset comprising 58 pairs of pre- and post-letrozole treatment specimens, pre- and post-letrozole specimens were analysed for Cdc6, Cdt1 and Orc1 expression. In this patient cohort, we found that expression level of Cdc6 was significantly lower post-letrozole than pre-letrozole (ANOVA test, p < 0.001; Fig. 7A). Similar results were observed for Cdt1 (ANOVA test, p = 0.003; Fig. 7B), but not for Orc1. In line with our *in vitro* experiments, inhibition of ER signalling resulted in reduced expression of Cdc6 and Cdt1.

Interestingly, the expression level of Cdc6 was significantly lower in those patients who responded to the letrozole treatment than those patients who did not respond to treatment in the pre-treatment specimens (post-hoc, Games-Howell test, p = 0.04; Fig. 7C). Similarly, there was a significantly higher expression of Cdc6 in the post-treatment specimens which responded to letrozole treatment than those which did not respond to the letrozole (post-hoc, Games-Howell test, p = 0.003; Fig. 7D). These results suggest that the expression level of Cdc6 may predict the responsiveness of the breast cancer to inhibition of ER signalling.

Importantly, the association between Cdc6 and patient survival was only observed in ER positive breast cancer but not in ER negative breast cancer. While there was no significant difference in survival time between patients with breast cancer expressing a high level of Cdc6 and those expressing a low level of Cdc6 in ER-negative patients (Wilcoxon-Gehan test, p = 0.420; Fig. 4D), a high level of Cdc6 expression was significantly associated with a shorter survival time in breast cancer patients with ER positive breast cancer (Wilconxon-Gehan test, p = 0.004; Fig. 4E). No such correlation was observed for Cdt1 or for Orc1. These results suggest that Cdc6 may play a more important role in ER positive breast cancer and that breast cancer patients with ER positive status and a low level expression of Cdc6 may have a better prognosis as a result of better response to ER inhibition.

## Discussion

Previously, we have shown that MCM2-7 genes, when overexpressed in breast cancer specimens confer a poor prognosis^16^. Since assembly of the pre-RC requires the presence of Cdc6 and Cdt1^4^, it is highly possible that overexpression of these two genes may also contribute to a poorer prognosis in breast cancer patients. Indeed, in the present study, we found that a high level expression of either Cdc6 or Cdt1 in the breast cancer specimens was associated with a shorter survival of the patients. Importantly, we observed a very strong correlation between the expression of Cdc6, Cdt1 and MCM2-7 genes. Our results suggest that these genes may cooperate in driving a more aggressive behaviour of breast cancer. In line with these results, Cdc6 and Cdt1 were expressed to a higher level in breast cancer cell lines than in normal breast epithelial cells, and they are suppressed by withdrawal of growth signal from the culture medium. Importantly, inhibition of ER signalling, which suppresses ER-positive breast cancer cell growth, decreased the expression of both Cdc6 and Cdt1 *in vitro* and in human breast cancer specimens, while lower expression of Cdc6 correlated with a better response of the tumour towards inhibition of ER signalling. In addition, we found that Cdc6 and Cdt1 expression levels were both higher in ER-negative breast cancer than ER-positive breast cancer, implying that increased Cdc6 and Cdt1 expression in ER- may contribute to increased aggressiveness; the results were also similar to MCM2-7 genes that we have previously described^16^.

It is well known that DNA replication is one very important step in cell proliferation, while increase in cell proliferation is one of the hallmarks in cancer development^22^. MCM2-7, Cdc6 and Cdt1 collaborate to regulate DNA replication, and each DNA replication licensing requires their sequential recruitment to the replication origin^1^. Increased expression of MCM confers poor prognosis in various cancer types, including breast cancer^16, 18^, lung cancer^23^, colon cancer^24^ and glioma^25^. However, the role of Cdc6 and Cdt1 in cancer development is less well understood. In the present report, we found that expression of Cdc6 and Cdt1 was positively correlated with MCM2-7 overexpression, while high levels expression of Cdc6 and Cdt1 were associated with poor prognosis in breast cancer patients. Importantly, both Cdc6 and Cdt1 expression was increased in breast cancer cells compared to normal cells, while their expression was upregulated by growth stimuli, such as FBS and estrogen, suggesting that these two genes are highly correlated with proliferation signal and may play an important role in promoting a more aggressive behaviour in breast cancer cells. However, since Cdc6, Cdt1 and Orc1 all play an important role in DNA replication licensing, the reasons for Orc1 mRNA expression not being a prognostic factor in breast cancer may be worth further investigation. Cdc6, or to a lesser extent Cdt1 but not that of Orc1, may be rate limiting factor in DNA replication licensing, which means that increased expression of Orc1 may not be resulting in increased rate of DNA replication. Otherwise, Orc1 may be regulated at its post-transcriptional or post-translational level instead of transcriptional level.

Estrogen has been shown to promote cell proliferation and enhance the expression of Cdc6 in cancer cells^14^, while previously, we have also demonstrated the association between ER and expression of MCM2-7^16^. In the present study, we have shown that both Cdc6 and Cdt1 expression was supressed by inhibition of ER signalling both *in vitro* and in patients, suggesting that these two genes are downstream targets of the ER. In addition, the prognostic power of Cdc6 only reached statistical significant in ER-positive but not in ER-negative breast cancer, suggesting that ER-positive breast cancer may be more sensitive to limited replicative licensing. However, whether these two genes are direct targets of ER requires further investigation. Interestingly, the degree of Cdc6 expression seemed to be correlatable with responsiveness of the cancer towards letrozole. This result strongly indicates that Cdc6 could also be a predictive biomarker for response to inhibition of estrogen receptor signalling therapy. Recent phase III clinical trials have shown that inhibition of HER2^26^ or mTOR^27^ when combined with ER inhibition produced a better therapeutic response/outcome than ER inhibition alone. As breast cancer with a high level expression of Cdc6 were less responsive to ER inhibition, it is tempting to speculate that the modality of combining inhibitions of ER and other pathways may be more beneficial to patients with high level of Cdc6, who respond less well to ER inhibition alone.

## Methods

### Extraction of clinical and microarray gene expression data from breast cancer patient datasets

Seven breast cancer patient datasets, GSE1456^28^, GSE2034^29^, GSE3143^30^, GSE3494^31^, GSE7390^32^, GSE11121^33^ and GSE12276^34^, with survival status available were included in our analyses; the combined dataset consisted of 1441 breast cancer patients; These cohorts were used in our previous study for investigating the prognostic significance of MCMs in breast cancer^16^. One further breast cancer dataset, GSE5462^35^, which comprises 58 pairs of pre- and post-letrozole treatment specimens and associated response data available was also included. The data from these datasets were extracted from the Gene Expression Omnibus (GEO) database as previously described^16^. The datasets used in the current study are all publicly available and the links to access these datasets are listed in Supplementary Table 1. Patients were divided into four groups based on the expression levels of genes of interest using upper quartile, median and lower quartile expression levels as the cut-off points. In Kaplan-Meier analysis where patients were divided into two groups, median expression level was used as the cut-off point.

### Statistical analysis for breast cancer datasets

The statistical analysis was performed using SPSS19.0. The associations between expression levels of genes were analyzed by Chi-Square test while the correlations between expression levels of genes were analyzed by Spearman’s rank test. The associations between expression levels of genes and survival were analyzed by Kaplan-Meier analysis compared by Wilcoxon-Gehen test. Univariate and Multivariate Cox-regression analyses were used to identify independent predictors for patient survival using a forward stepwise approach with an entry limit of p < 0.05. The difference in the expression levels of genes of interest pre- or post-letrozole treatment was compared by repeated measure ANOVA, while the difference in expression level of genes in specimens with different response to letrozole was compared by ANOVA. Results were considered significant when p-value was smaller than 0.05.

### Cell Culture and treatment

The human breast carcinoma cell lines (MCF7, MCF10A, MDA MB231) were purchased from American Type Culture Collection (ATCC, Manassas, VA, USA). MCF7 and MDA MB231 cells were maintained in Dulbecco’s minimal essential medium (DMEM) (Gibco) supplemented with 10% fetal bovine serum (FBS, Gibco), 4 mM L-glutamine (Gibco), 1% penicillin-streptomycin (Gibco), and sodium pyruvate (1 mM). For MCF7 cells, medium was as above, with the addition of 0.01 mg/ml bovine insulin (Sigma). MCF10A cells were maintained in DMEM medium supplemented with 5% FBS (Gibco), 1% penicillin-streptomycin (Gibco), 20 ng/ml EGF (Peprotech), 0.5 ug/ml hydrocortisone (Sigma), 100ng cholera toxin (Sigma) and 10 ug/ml bovine insulin (Sigma). All cells were cultured at 37 °C in a humidified atmosphere of 95% air and 5% CO_2_. For serum starvation, cells were cultured in respective media without FBS and without phenol red.

For tamoxifen treatment, MCF7 cells were treated with 18 uM tamoxifen (Sigma). The cells were harvested at 0, 12, 24 hours and protein and gene expression was quantified by Western blot and qRT-PCR respectively.

### Western blot

Cells were harvested using a cell scraper and suspended in RIPA buffer (150 mM NaCl; 5 mM EDTA, 50 mM Tris, 1% Triton X-100, 0.5% sodium deoxycholate, 0.1% SDS) supplemented with protease inhibitor (Roche) and phosphatase inhibitor (Roche). Endogenous proteins from whole cell extracts were isolated by sonication followed by centrifugation. Proteins were quantified using BCA protein assay (ThermoFisher Scientific) and equal amounts of proteins were loaded onto 4–12% SDS–polyacrylamide gel electrophoresis (SDS–PAGE) gels and electrophoretically transferred to a PDVF membrane using Novex iBlot transfer stack (ThermoFisher Scientific) on a iBlot gel transfer device (ThermoFisher Scientific). The membrane containing the transferred protein was blocked with 5% BSA at room temperature for 1 h. Target proteins were detected by incubating the membrane at 4 °C overnight with primary anti-Cdt1 antibody (#8064 Cell Signaling Technology) (1:500) or anti-Cdc6 antibody (#3387 cell signaling technology) (1:500) and primary anti-β-actin antibody (Santa Cruz) (1:5000). Blot was further developed using horseradish peroxidase (HRP)-conjugated secondary antibodies (1:5000) (Santa Cruz) and detected using Immobilon Western Chemilum HRP substrate (Merck). Blots were visualized on a ChemiDoc Touch Imaging System (BioRad).

### Quantitative RT-PCR

Cells were harvested by cell scraping in RLT lysis buffer and total RNA was isolated using RNeasy Mini Kit (Qiagen). RNA quantity and quality were measured using a Nanodrop™ spectrophotometer (NanoDrop products). First strand cDNA synthesis was performed from 1 μg total RNA using a High Capacity cDNA Reverse Transcription Kit (Applied Biosystems) on a BioRad C1000 Touch^TM^ Thermal Cycler. qRT-PCR analysis was performed using TaqMan® Universal PCR Master Mix (Applied Biosystems) and Taqman probes specific for Cdc6 (HS00154374_m1), Cdt1 (HS00368864_m1) and GAPDH (Hs02758991_g1) purchased from ThermoFisher Scientific. The analysis was performed using a Mx3005 P qPCR System (Agilent) thermocycler. The differences observed between the control and treated groups for qRT-PCR were analyzed by unpaired Student’s t-test (two-tailed) using GraphPad Prism 6 (GraphPad Software). The results were expressed as the Mean ± SEM (standard error of mean) from three different replicates and a value of p < 0.05 was considered statistically significant.

## Electronic supplementary material

Supplementary Table 1
