# Supplementary material for: The prognostic significance of Cdc6 and Cdt1 in breast cancer
Source: Sci Rep. 2017 Apr 20;7:985. doi: 10.1038/s41598-017-00998-9 (PMC5430515; doi:10.1038/s41598-017-00998-9)
Supplement: Supplementary file 1 — Supplementary Table 1 [file 41598_2017_998_MOESM1_ESM.pdf]

The prognostic significance of Cdc6 and Cdt1 in breast cancer

Ravikiran Mahadevappa[1,#], Henrique Neves[1,#], Shun Ming Yuen[1], Yuchen Bai[1], Cian M. McCrudden[2], Hiu Fung Yuen[3], Qing Wen[4], Shu Dong Zhang[5] and Hang Fai Kwok[1,*]

[1]Faculty of Health Sciences, University of Macau, Avenida de Universidade, Taipa, Macau SAR

[2]School of Pharmacy, Queen's University Belfast, Belfast, United Kingdom

[3]Institute of Molecular and Cell Biology, A*STAR, Singapore

[4]Centre for Cancer Research & Cell Biology, School of Medicine, Dentistry and Biomedical Sciences, Queen's University of Belfast, Belfast, United Kingdom

[5]Northern Ireland Centre for Stratified Medicine, Biomedical Sciences Research Institute, University of Ulster, Londonderry, United Kingdom

Supplementary Table 1. The datasets used and its accessible links in the current study

Dataset ID	Website for access
GSE1456	https://www.ncbi.nlm.nih.gov/geo/query/acc.cgi?acc=GSE1456
GSE2034	https://www.ncbi.nlm.nih.gov/geo/query/acc.cgi?acc=GSE2034
GSE3143	https://www.ncbi.nlm.nih.gov/geo/query/acc.cgi?acc=GSE3143
GSE3494	https://www.ncbi.nlm.nih.gov/geo/query/acc.cgi?acc=GSE3494
GSE7390	https://www.ncbi.nlm.nih.gov/geo/query/acc.cgi?acc=GSE7390
GSE11121	https://www.ncbi.nlm.nih.gov/geo/query/acc.cgi?acc=GSE11121
GSE12276	https://www.ncbi.nlm.nih.gov/geo/query/acc.cgi?acc=GSE11121
GSE5462	https://www.ncbi.nlm.nih.gov/geo/query/acc.cgi?acc=GSE5462
